# Swallowing after non-surgical treatment (radiation therapy / radiochemotherapy protocol) of laryngeal cancer

**DOI:** 10.1590/S1808-86942011000100016

**Published:** 2015-10-19

**Authors:** Juliana Portas, Claudia Pereira Socci, Eliana Perissato Scian, Débora dos Santos Queija, Alessandra Sampaio Ferreira, Rogério Aparecido Dedivitis, Ana Paula Brandão Barros

**Affiliations:** 1 Fundação Antonio Prudente, São Paulo, Brazil. Clinical speech therapist; 2 Higher training in speech therapy applied to neurological rehabilitation, UNIFESP-EPM, São Paulo, Brazil. Hospital speech therapist; 3 Specialized in auditory processing, USP, São Paulo, Brazil. Clinical speech therapist; 4 Graduate course at the Heliópolis Hospital (HOSPHEL), São Paulo, Brazil. Clinical speech therapist; 5 Speech therapy graduate, clinical speech therapist; 6 Associate professor, Fundação Lusíada, Unilus, Santos. Professor of the graduate course in health sciences, Heliópolis Hospital; 7 Graduate course at the Fundação Antônio Prudente (FAP), São Paulo, Brazil. Head of the Voice Rehabilitation and Swallowing Unit, Heliópolis Hospital, São Paulo, Brazil

**Keywords:** deglutition disorders laryngeal neoplasms, radiotherapy, combined modality therapy

## Abstract

Radiation therapy and radiochemotherapy protocols can cause swallowing difficulties.

**Aim:** To evaluate swallowing in patients undergoing radiation therapy and radiochemotherapy protocol only for the treatment of laryngeal tumors.

**Methods:** A prospective study of 20 patients, with a mean age of 62 years, at the end of oncological therapy. Six patients (30%) underwent radiation therapy, and 14 patients (70%) underwent combined therapy. The mean time between treatment and an evaluation of swallowing was 8.5 months. Videofluoroscopy was done to assess the preparatory, oral and pharyngeal phases of swallowing.

**Results:** All patients had only an oral diet. Normal swallowing was present in only 25% of patients. The swallowing videofluoroscopic examination identified the following changes: bolus formation (85%), bolus ejection (60%), oral cavity stasis (55%), changes in the onset of the pharyngeal phase (100%), decreased laryngeal elevation (65%), and hypopharyngeal stasis (80%). Laryngeal penetration was observed in 25% of the cases; 40% presented tracheal aspiration. The grade of penetration/ aspiration was mild in 60% of cases. Aspiration was silent in 35% of patients. Although 75% of patients had dysphagia, only 25% complained of swallowing difficulties.

**Conclusion:** Patients with laryngeal cancer that underwent radiation therapy/combined treatment can present changes in all swallowing phases, or may be asymptomatic.

## INTRODUCTION

Treatment for initial or advanced head and neck tumors may include radiotherapy alone or associated with surgery and chemotherapy.^1^ The main purpose of non-surgical oncologic therapy is to preserve breathing, swallowing and communication fuctions.^2,^^3^

A current debate is the impact of this type of therapy on laryngeal function and quality of life, since treatment may cause malnutrition, dehydration, weight loss, pain, dysphonia, dysphagia and ototoxicity.^4^ Radiotherapy alone may cause several dysfunctions in different degrees, such as: xerostomy, odynophagia, weight loss, and a need for alternative feeding routes.^5^

Disordered swallowing affects feeding efficiency and safety; regardless of severity, the quality of life and several lifestyle aspects may be compromised.^6^

Late functional results were analyzed based on videofluoroscopy swallow studies of 31 subjects after the end of combined radiotherapy and chemotherapy. None of these patients had been treated with swallowing therapy at the time of evaluation. Diagnoses were made of the preparatory, oral, and pharyngeal phases of swallowing. Swallowing was considered functional in 35.5% of patients; mild to moderate dysphagia was found in 54.8% of the sample, and severe dysphagia was encountered in 9.6% of patients.^7^

The impact of chemoradiotherapy on swallowing, nutrition and quality of life of 59 patients was assessed. Of these, 23 patients underwent a videofluoroscopy swallow study 3.5 months after the end of chemoradiotherapy. Tracheal aspiration was found in 78% of these patients, 35% of which were silent.^8^

Another study assessed the severity of aspiration in 63 patients after radiotherapy and chemotherapy and found that 59% had aspiration, of which 33% were severe cases. These authors also noted a 9.5% death rate in patients that presented pneumonia.^9^

Still another paper evaluated 26 patients after intensity-modulated chemoradiation therapy at three moments: immediately after, three months after, and 12 months after the treatment. The authors found decreased tongue retraction, uncoordinated swallowing, decreased laryngeal elevation and closure, inversion of the epiglottis, increased pharyngeal transit time, and silent aspiration.^10^

Some studies have found significant worsening of some videofluorographic swallow study parameters three and 12 months after the end of therapy.^11,^^12^

An understanding of the functional results of swallowing is necessary. Thus, the purpose of this study was to characterize swallowing in patients with laryngeal tumors treated with radiotherapy only or combined with chemotherapy by using videofluoroscopic swallow studies.

## MATERIAL AND METHOD

A prospective study was made of 20 patients with laryngeal tumors who had been treated with radiotherapy alone or combined with chemotherapy at a Head & Neck Unit of an institution. Patients were enrolled based on the following inclusion criteria: patients having undergone radiotherapy only or combined with chemotherapy for the treatment of laryngeal tumors; at least a two-month interval after the end of oncologic therapy; signing a free informed consent form to participate. The exclusion criteria were: active disease; having had head and neck surgery; a history of neurological conditions. These factors could themselves alter swallowing. All patients underwent a videofluoroscopic swallow study.

A Philips, Chalanger® N 800 HF model radiology equipment was used to characterize swallowing. The exam consisted of analyzing the swallowing process from the lateral and anterior-posterior views while the patient swallowed 20 ml of liquid, 20 ml of a semi-liquid product, 15 ml of a semi-solid product, and part of a cookie. The events of the preparatory and oral phases were assessed: formation of the bolus, mobility of phonoarticulatory organs, ejection of the bolus, stasis in the oral cavity; contact between the tongue and the palate; identifying the site of the beginning of the pharyngeal phase of swallowing; and the pharyngeal phase (elevation of the larynx, stasis in the oropharynx and in the hypopharynx). The tests were based on the penetration and aspiration scale proposed by Rosenbek et al.^13^ (Table 1) and classified according to the severity of dysphagia scale proposed by O'Neil et al.^14^ (Table 2). The tests were show three times and evaluated by consensus between three speech therapists with at least three years experience in interpreting videofluoroscopic swallow studies of treated oncologic patients. The statistical analysis consisted of a frequency distribution and central tendency and variability measures for numerical values.

**Table 1:** Penetration and aspiration scale - Rosenbek et al., 1996.

Category	Score	Description
PENETRATION	1	Contrast does not enter the airway
	2	Contrast enters the airway; remains above local folds, no residue
	3	Contrast remains above vocal folds, visible residue remains
	4	Contrast contacts vocal folds, no residue
	5	Contrast contacts vocal folds, visible residue remains
	6	Contrast passes glottis, no sub-glottic residue visible
ASPIRATION	7	Contrast passes glottis, visible sub-glottic residue despite patient's response
	8	Contrast passes glottis, visible sub-glottic residue, absent patient response

**Table 2:** Severity of dysphagia - O'Neil et al., 1999

**Severity of dysphagia**
Oral route (normal diet)
Level 7 - normal in all situations
Level 6 - within functional limits
**Modified oral route**
Level 5 - mild dysphagia
Level 4 - mild-moderate dysphagia
Level 3 - moderate dysphagia
**Nothing per orum**
Level 2 - moderately-severe dysphagia
Level 1 - severe dysphagia

## RESULTS

The study sample comprised 20 patients with a mean age of 62 years, all of which had undergone treatment for laryngeal tumors. Six of these (30%) were treated with radiotherapy alone, and 14 patients (70%) were treated with chemoradiotherapy. The mean time between medical treatment and the phonoaudiological assessment was 8.5 months. Initial tumors (T1 and T2) were found in 55% of patients, and advanced (T3 and T4) tumors were present in 45% of cases; 75% were classified as N0. The time elapsed between the end of oncologic treatment and testing for this study ranged from 2 to 60 months (Table 3).

**Table 3:** Demographic, medical and treatment features

Features	Variables	Measurement (N=20) and Frequency (%)
Gender	Male	16 (80)
	Female	4 (20)
Age	Minimum-Maximum	48-80
	Median	61
	Mean ± standard deviation	62,3±8,89
Site of the lesion	Glottis	8 (40)
	Supra-glottis	12 (60)
T	1	1 (5)
	2	10 (50)
	3	7 (35)
	4	2 (10)
N	0	15 (75)
	1	3 (15)
	2	2 (10)
Treatment	Radiotherapy alone	6 (30)
	Chemoradiotherapy	14 (70)
Time after treatment (months)	Minimum-Maximum	2-60
	Median	3
	Mean ± standard deviation	8,5±13,7

Table 4 shows the classification of dysphagia results.

**Table 4:** Functional results of swallowing - O' Neil et al., 1999

Severity of dysphagia	Measurement (N=20) and Frequency (%)
**Oral route (normal diet)**	
Level 7 - normal in all situations	0
Level 6 - within functional limits	5 (25)
**Modified oral route**	
Level 5 - mild dysphagia	6 (30)
Level 4 - mild-moderate dysphagia	8 (40)
Level 3 - moderate dysphagia	0
**Nothing per orum**	
Level 2 - moderately-severe dysphagia	1 (5)
Level 1 - severe dysphagia	0

All patients had exclusively oral diets at the time of the swallow study. Table 5 presents the findings of the videofluoroscopic swallow studies, showing that 80% of the sample had stasis in the hypopharynx and 100% had stasis in the oropharynx.

**Table 5:** Preparatory, oral and pharyngeal phases of swallowing

Variables	Categories	Total Measurement (N=20) and Frequency (%)
Preparatory and oral phases		
Forming the bolus	Adequate	3 (15)
	Inadequate	17 (85)
Motility of phonoarticulatory organs	Adequate	16 (80)
	Inadequate	4 (20)
Ejection of bolus	Adequate	8 (40)
	Inadequate	12 (60)
Stasis in the oral cavity	Absent	9 (45)
	Mild	7 (35)
	Moderate	4 (20)
	Severe	0
Contact of tongue with pharynx	Adequate	15 (75)
	Inadequate	5 (25)
Pharyngeal phase		
Beginning of the pharyngeal phase	Vallecula	18 (90)
	Pharyngo-esophageal transition	2 (10)
Elevation of the larynx	Adequate	7 (35)
	Mild reduced	9 (45)
	Moderate reduced	4 (20)
Stasis in oropharynx	Absent	0
	Mild	5 (75)
	Moderate	3 (15)
	Severe	2 (10)
Stasis in hypopharynx	Absent	4 (20)
	Mild	12 (60)
	Moderate	2 (10)
	Severe	2 (10)

No food entered the airways in 35% of cases. Laryngeal penetration was diagnosed in 25% of cases; penetration followed by tracheal aspiration was found in 40% of cases. The degree of penetration/aspiration was mild in 60% of cases; however, aspiration was silent in 35% of the sample (Table 6). Only 25% of patients complained of swallowing difficulties in the clinical history taken before videofluoroscopy.

**Table 6:** Penetration and aspiration scale and level, and severity of dysphagia

Variables	Categories	Total Measurement and Frequency (%)
Penetration level	1	7 (35)
	2	2 (10)
	3	2 (10)
	4	1 (5)
	5	0 (0)
	6	1 (5)
Aspiration level	7	0 (0)
	8	7 (35)
Penetration/aspiration level	Absent	7 (35)
	Mild	12 (60)
	Moderate	1 (5)

Enteral nutrition was used in 10% of cases; the mean time elapsed with the nasoenteric tube in place was four months. Tracheostomy was carried out in 15% of the sample during the treatment; the mean duration of tracheostomy was 6 months. Bronchopneumonia was present in 5 patients (25%) during the treatment. Of these, 80% had tracheal aspiration during the videofluoroscopy swallow test.

## DISCUSSION

The functional results of swallowing in the study sample revealed that most of these patients had some degree of dysphagia. All patients had exclusively oral diets, 75% of the sample had some degree of dysphagia, but only 25% of cases complained about swallowing.

Using specific tools for evaluating dysphagia revealed changes in all phases of swallowing, explained as sequelae of treatment. All preparatory and oral phase events that were studied were altered; in most cases, food bolus formation was affected. Cintra et al.^7^ noted that patients treated with chemoradiation may suffer effects on the oral phases of swallowing, even in cases of laryngeal tumors only.

The disorders that were found may be explained as complications of radiotherapy.^15^ Radiotherapy causes inflammation that manifests as mucositis, dermatitis, soft-tissue edema, increased production of mucus, and xerostomy; as late manifestations, fibrosis and tissue rigidity occur because of hypoxia and chronic oxidation.

Xerostomy, increased production of mucus, and restricted mobility of phonoarticulatory organs may affect food bolus formation, which in turn will not be ejected adequately; the result is food stasis in the oral cavity. Tissue edema and fibrosis, even when the radiation field is not the mouth, may result in decreased mobility amplitude of phonoarticulatory organs, resulting in altered pharyngeal, oral and preparatory phases.

The main finding during the pharyngeal phase was decreased laryngeal elevation - the full hyolaryngeal excursion does not take place during swallowing. This event may be seen in the presence of post-therapy tracheostomy, edema and laryngeal fibrosis.^5,^^7,^^10^, ^11^, ^12^

Xerostomy and mucositis associated with decreased laryngeal elevation may lead to food stasis in the oral cavity and hypopharynx. Food residue after swallowing reflects changes in the mobility of phonoarticulatory organs during the preparatory and oral phases; this may result in additional changes in subsequent phases.

Our data showed that 40% of the sample presented tracheal aspiration, 35% of which was silent. Murphy & Gilbert^15^ have stated that silent aspiration is frequent in irradiated patients and that the cough reflex is ineffective or absent in at least half of these patients. Studies in the literature have underlined the importance of objective examinations of these patients, as tracheal aspiration may result in bronchopneumonia and even death. Nguyen et al.^9^ assessed 63 patients treated with chemoradiation and found severe aspiration in 33% of cases, and 9% of deaths because of bronchopneumonia. Hutcheson et al.^12^ reported post-treatment tracheal aspiration in 84% of cases.

As complications of therapy, 15% of cases required tracheostomy, 10% of cases required enteral nutrition, and 25% of cases had bronchopneumonia during and/or following the treatment; only one patient, however, was classified as having severe dysphagia.

Tracheostomy may be required because of radiotherapy-associated edema, which may occlude the glottis and obstruct airflow. Tracheostomy is also a risk factor for dysphagia because it may result in sensory and mechanical changes in the hyolarynx.

Nguyen et al.^9^ reported that 100% of their study sample required enteral nutrition at some point during the first three years of treatment. This alternative route for nutrition may be required to avoid malnutrition because of decreased oral intake of food in patients with frequent aspiration.

Hutcheson et al.^12^ reported that 78% of their study sample required enteral tubes at some point during treatment; in 52% of cases, it was the alternative route for feeding the patients. These authors suggested that the degree of dysphagia and the dependence on the tube after the end of treatment were predictive factors for aspiration, as opposed to the period before treatment, when enteral nutrition is used to provide adequate nutrition to patients.

Pauloski et al.^16^ studied 170 patients treated with chemoradiation and correlated the changes in dysphagia. They found that decreased laryngeal elevation and opening of the pharyngo-esophageal transition correlated significantly with oral intake or food restriction because of consistency.

A few authors have suggested that the effects of chemotherapy and radiation may reinforce each other, interfering further with swallowing. Greeven et al.^17^ found that in patients with stage T3 and T4 tumors, 21% required a gastrostomy after radiotherapy alone, while 77% required this procedure after chemoradiation.

Although 75% of the sample had some degree of dysphagia, only 25% of patients had complaints about swallowing.

Papers in the literature have shown that chemoradiotherapy does not necessarily preserve laryngeal function during swallowing. Silent aspiration may occur regardless of whether patients complain or not about swallowing. Thus, phonoaudiological assessments and monitoring, and objective swallowing tests, are important for identifying sequelae of oncologic treatment.

In the present study we did not correlate the results of radiotherapy alone with chemoradiation. Additional studies with larger series are suggested for evaluating the functional results of swallowing and their correlation with the type of treatment.

## CONCLUSION

Laryngeal cancer patients treated with radiotherapy/ chemoradiotherapy may present changes in all phases of swallowing, regardless of complaints about swallowing.
